# Prognostic significance of inflammation-based prognostic scoring in patients with upper urinary tract urothelial carcinoma

**DOI:** 10.1590/S1677-5538.IBJU.2018.0251

**Published:** 2019-07-27

**Authors:** Taisuke Suyama, Shigeki Kanbe, Masanobu Maegawa, Hirofumi Shimizu, Koichi Nakajima

**Affiliations:** 1 Department of Genitourinary, Sanaikai General Hospital (IMS), Japan; 2 Department of Genitourinary, Toho University Omori Medical Center, Japan

**Keywords:** C-Reactive Protein, Keratin-19, Biomarkers, Urinary Tract

## Abstract

**Objectives:**

To investigate whether Glasgow Prognostic Score has prognostic significance in patients with upper urinary urothelial carcinoma.

**Patients and methods:**

We retrospectively reviewed the clinical records of 74 patients with upper urinary urothelial carcinoma. We set the cut-off value for C-reactive protein as 1.0mg/dL, and 3.5mg/dL for albumin as Glasgow Prognostic Score. Their blood data including albumin and C-reactive protein for Glasgow Prognostic Score and cytokeratin 19 fragment 21-1 as a tumor marker were measured before starting treatment. The patients were stratified into three groups with Glasgow Prognostic Score: The Group-1, albumin ≥3.5g/dL and C-reactive protein < 1.0mg/dL; Group-2, albumin < 3.5g/dL or C-reactive protein ≥1.0mg/dL; Group-3, albumin < 3.5g/dL and C-reactive protein ≥1.0mg/dL.

**Results:**

The median follow-up for all patients was 26.9 months (range: 10.9-91.1 months), during which 37 (50%) patients died. There was a significant difference in the estimated survival rate among the 3 groups stratified by Glasgow Prognostic Score. The estimated survival rate in the Group-1 was significantly higher than those in Groups 2 and 3.

In the univariate analysis C-reactive protein, serum cytokeratin 19 fragment 21-1 and Glasgow Prognostic Score were significant predictors of overall survival. On the multivariate analysis, serum cytokeratin 19 fragment 21-1 and Glasgow Prognostic Score were independently associated with shorter overall survival.

**Conclusion:**

Our review suggests Glasgow Prognostic Score may play as a prognostic predictor for upper urinary urothelial carcinoma.

## INTRODUCTION

Urothelial carcinoma (UC) is the most common histological type of urinary system malignancy. UC arises from the urothelium of the entire urinary tract, from the renal pelvis and ureter to the urinary bladder and urethra.

The incidence of upper urinary urothelial carcinoma (UTUC) is relatively low, comprising only 5-10% of all urothelial malignancies (1). Although all UC shares some same cancer-causing materials including the occupation revelation to a cigarette and aromatic amine (2), some diseases have a closer relationship with UTUC. However, the prognosis for UTUC is generally poorer than that for bladder cancer (3); clinically effective prognostic predictors with a high specificity for UTUC has not been established such as prostate specific antigen (PSA) for prostate cancer and a-fetoprotein (AFP) or human chorionic gonadotropin (hCG) for testicular tumors.

Thus, preoperative staging of UTUC is not simple, and radical nephroureterectomy (RNU) with bladder-cuff removal is traditionally considered the standard of care for localized disease (4). However, recent advances in diagnostic imaging and endoscopic armamentarium (5) have markedly enhanced the role of kidney-sparing surgery for well-selected patients in the latest European Association of Urology (EAU) guidelines (4).

Regarding convalescence, the 5-year cancer-specific survival (CSS) reaches 50% for locally muscle invasive tumors (pT2-3), but within 10% for progressive disease (pT4) (6).

Serum C-reactive protein (CRP) is well known to be synthesized by the liver in response to inflammation. Several inflammation-based scoring systems, Glasgow Prognostic Score (GPS) and neutrophil-to-lymphocyte ratio (NLR) have been reported as useful prognostic indicators of several types of malignancies in the field of surgery (7–10). GPS is based on the combination of CRP and albumin levels and helps it as a significant prognostic factor in patients with cancer, reflecting both inflammatory components and nutritional status.

Originally, the concept of GPS was created to evaluate elevated CRP concentration and the effect of low albumin on the survival rate in patients with advanced lung cancer (11).

Also, since we reported that CYFRA 21-1 could be a biomarker of UTUC before (12), we are considering adding CYFRA 21-1 also in this study.

A preoperative expectation of the prognosis in patients can play an important role in selecting the appropriate treatments. Herein, we performed a retrospective study to investigate whether GPS has prognostic significance in patients with UTUC.

A committee appointed to consider ethical issues approved this study on December 18^th^, 2017.

## PATIENTS AND METHODS

### Study design

The present study targeted patients who were operated due to upper urinary tract urothelial carcinoma (UTUC) from April 1990 to January 2016 at Toho University Hospital and Sanaikai General Hospital, and diagnosed with UTUC by imaging (mostly computed tomography scan) and urine cytology. A total of 74 patients were diagnosed with UTUC.

Their blood data including albumin and CRP for GPS and cytokeratin 19 fragment 21-1 (CYFRA 21-1) as a tumor marker were measured basically two days before surgery, excluding diseases that are strongly expected to raise CRP, such as urinary tract infection.

The patients were stratified into three groups with Glasgow prognostic score (GPS): The Group-1, albumin ≥ 3.5g/dL and CRP < 1.0mg/dL; Group-2, albumin < 3.5g/dL or CRP ≥ 1.0mg/dL; Group-3, albumin < 3.5g/dL and CRP ≥ 1.0mg/dL.

And the performance status of all target patients was 0 or 1.

### Statistical analysis

Data were expressed as numbers with percentage or means ± standard deviation (SD) or medians with quartile values (25%-75%). The differences in continuous variables between the two groups were compared by unpaired t-test or Mann-Whitney test per the data distribution (normal or not). The differences in categorical variables between the two groups were investigated by Fisher's exact test. The Kaplan-Meier method was used to estimate survival distributions, and the differences between the two groups were compared using the log-rank test. The Cox proportional hazard regression model analysis was performed to identify the predictors for overall deaths following the operation of UTUC and to calculate hazard risk (HR) and 95% confidence intervals (95% CI). Univariate and multivariate Cox regression analyses were performed with and without adjustment for other variables. Because of comparisons between death and alive groups, variables of p < 0.1 were selected and adopted for Cox proportional hazard regression model. In the case there were collinearity characteristics between 2 variables, either of two variables was excluded from the multiple regression model. Receiver operating characteristics (ROC) curve analysis was used to determine the cut-off value of continuous parameters as a predictor for overall death. Also, the cut-off value of the predictor of total mortality was calculated using the area under the curve (AUC) and 95% CI. All statistical analyses were performed using JMP (R) version 12.2.0, and P-values < 0.05 were considered statistically significant.

## RESULTS

### Baseline charctaeristics

Table-1 shows characteristics of follow-up patients with UTUC. Univariate analysis of the background factors in the groups, CRP, GPS and CYFRA 21-1 were significantly different between two groups. And CRP and CYFRA 21-1 were significantly higher in the dead group. Other factors did not significantly differ.

**Table 1:** Characteristics of Follow-Up Patients with Urothelial Carcinoma.

	All cases N = 74	Overall Death N = 37	Alive N = 37	*P* values
Age, years	71.7±9.2	72.6±8.4	70.8±9.9	0.39 1)
Male	43 (58.0)	20 (54.1)	23 (62.2)	0.63 2)
BMI, kg/m^2^	21.2±2.6	21.3±3.2	21.2±2.0	0.76 1)
Albumin, g/dL	3.98±0.45	3.98±0.50	3.99±0.41	0.86 1)
WBC, /μL	7368±2330	7828±2437	6907±02153	0.089 1)
Platelet, x10^3^/μL	218±68	227±78	210±55	0.280 1)
NLR	4.77 [3.59-6.10]	4.82 [2.70-7.39]	4.74 [3.74-5.43]	0.717 3)
CRP, mg/dL	0.20 [0.10-0.70]	0.50 [0.10-1.00]	0.10 [0.10-0.30]	0.002 3)
GPS, 1/2/3	55/15/4	22/11/4	33/4/0	0.003 3)
CYFRA, ng/mL	5.97 [1.75-12.27]	6.13 [3.50-30.67]	2.30 [1.50-6.13]	<0.001 3)
Follow-up, month	26.9 [10.9-91.1]	16.0 [8.8-50.3]	50.7 [22.7-97.0]	0.005 3)

Data are expressed as means ± standard deviation or numbers with percentage or medians [25%-75% quartile value]. P values were determined by

1) unpaired t test,

2) Fisher's exact test,

3) Mann-Whitney test. BMI indicates body mass index; GPS, Glasgow prognostic score (1: Albumin ≥3.5 g/dL and CRP <1.0 mg/dL, 2: Albumin <3.5 g/dL or CRP ≥1.0 mg/dL, 3: Albumin <3.5 g/dL and CRP ≥1.0 mg/dL).

### Os associated with clinical parameters

**Figure 1:**
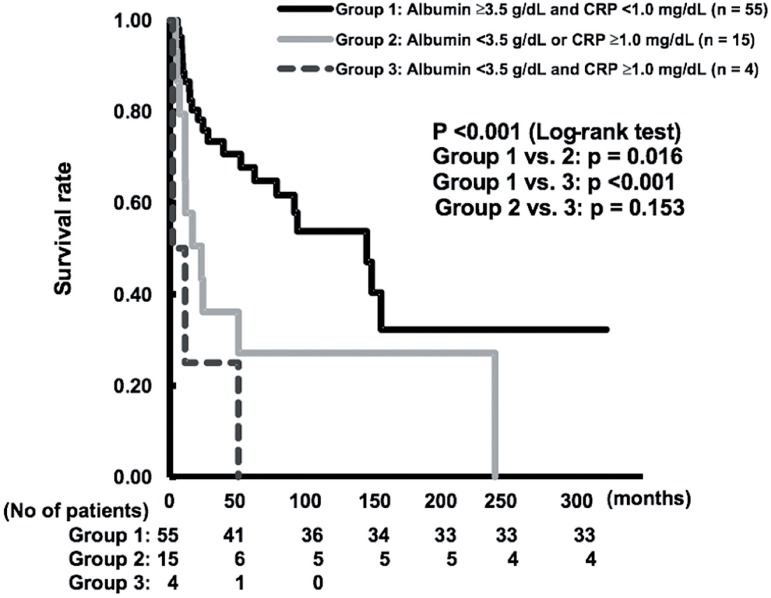
The Kaplan-Meier curves indicating overall survival rate among the 3 groups stratified by GPS.

The median follow-up in this series was 26.9 months (range; 10.9-91.1 months), and 37 (50%) patients died during follow-up (Table-1). Figure-1 shows there was a significant difference in the estimated survival rate among the 3 groups stratified by GPS (p < 0.001 as determined by the Log-rank test). The estimated survival rate in the Group-1 was significantly higher than those in the Groups-2 and 3 (p=0.01 and p < 0.001, respectively, no adjustment for multiple comparison).

Table-2 shows predictors for overall death in the patients with UTUC before and after adjustment with variables as determined by Cox proportional hazard regression model analysis. In the univariate analysis CRP (HR=3.26, 95% CI: 1.64 6.48, p=0.001), serum CYFRA 21-1 (HR=2.01, 95% CI: 1.02 – 3.96, p < 0.001) and GPS (HR=2.54, 95% CI: 1.55-4.14, p < 0.001) were significant predictors of overall survival.

**Table 2:** Predictors for Overall Death in the Patients with Urothelial Carcinoma before and after Adjustment with Variables Determined by Cox Proportional Hazard Regression Model Analysis.

Variables	Univariable Analysis		Multivariable Analysis	
	HR (95%CI)	*P* values	HR (95%CI)	*P* values
Age ≥70 years	2.02 (1.03 – 3.97)	0.04	1.76 (0.86 – 3.58)	0.12
Male	0.85 (0.45 - 1.64)	0.63	1.14 (0.58 - 2.24)	0.71
Albumin <3.5 g/dL	1.91 (0.78 - 4.64)	0.15	NA	
CRP ≥1.0 mg/dL	3.26 (1.64 - 6.48)	0.001	NA	
CYFRA ≥6.0 mg/dL*	2.01 (1.02 – 3.96)	<0.001	2.07 (1.05 – 4.08)	0.03
GPS	2.54 (1.55 – 4.14)	<0.001	2.28 (1.33 – 3.91)	0.003
Group 1	reference (1.00)		reference (1.00)	
Group 2	2.82 (0.88 - 9.06)	0.08	3.14 (0.88 - 11.16)	0.07
Group 3	6.68 (2.24 - 19.93)	0.001	6.18 (1.85 - 20.60)	0.003

HR indicates hazard risk; CI, confidence interval; NA, not apply and GPS indicates Glasgow prognostic score; 1: Albumin ≥3.5 g/dL and CRP <1.0 mg/dL, 2: Albumin <3.5 g/dL or CRP ≥1.0 mg/dL, 3: Albumin <3.5 g/dL and CRP ≥1.0 mg/dL.

* The median value in all patients.

On the multivariate analysis, serum CYFRA 21-2 (HR=2.07, 95% CI: 1.05-4.08, p=0.03) and GPS (HR=2.28, 95% CI: 1.33-3.91, p=0.003) were independently associated with shorter overall survival.

**Figure 2:**
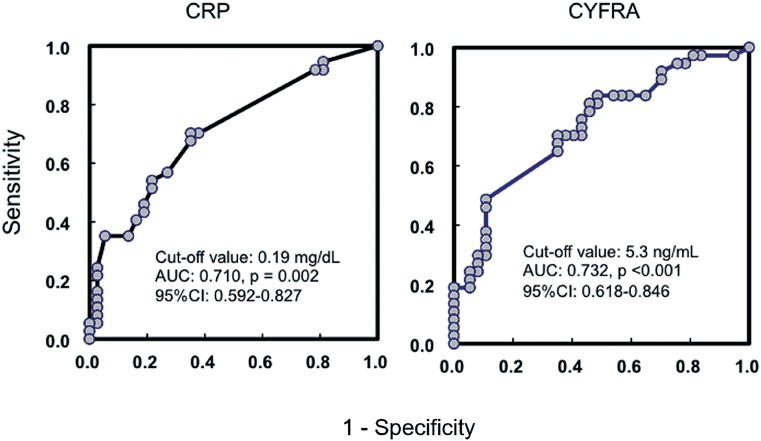
Receiver operating characteristics (ROC) curve, and the area under the curve (AUC) and 95% CI for determining the cut-off values of predictors for overall death.

And the cut-off value of CRP and CYFRA 21-1 were 0.19ng/dL and 5.3ng/mL, AUC were 0.710 and 0.732, 95% CI were 0.592-0.827 and 0.618-0.846 and P-values were 0.002 and < 0.001, respectively (Figure-2).

## DISCUSSION

GPS was proposed by Forrest et al., and so far, in non-small cell lung cancer (11), colon cancer (13), breast cancer (14), pancreas cancer (15), liver cancer (16), esophageal cancer (17) and it is reported to be a risk factor for long-term prognosis of various carcinomas.

Even in the urinary field, there are reports in kidney cancer (8, 18), prostate cancer (19), and bladder cancer (20).

GPS is calculated by two inspections, Albumin (Alb) and CRP which are generally measured in Japan. Therefore, it is particularly useful to evaluate easily and without using special inspection equipment. CRP is produced by interleukin (IL)-6 produced by macrophages and adipocytes, acting on hepatocytes. It is generally known that the CRP value correlates with the IL-6 concentration in the blood. It has also been found that elevated CRP in cancer patients correlates with the expression of IL-6 and IL-6 receptors in cancer cells in the absence of apparent inflammation. In other words, it is said that the grade of malignancy is high when there is a large number of IL-6 producing cells (21).

Albumin (alb) is a protein synthesized in the liver and present most in blood. Since Alb is also used as a source of amino acids, it is also used as an indicator of nutritional status. However, when inflammation is accompanied, protein production in the liver is inclined to the production of acute-phase proteins including CRP, accompanied by stress-induced hypoalbuminemia (22), which results in decreased production of Alb. Alb is used as an indicator of nutrition. However, it is pointed out that there is a problem there (23, 24). In addition, there are liver diseases such as protein-losing enteropathy and nephrotic syndrome that the production of Alb itself deteriorates. However, by correcting this with CRP in GPS, Alb increases credibility as an indicator of nutrition. Therefore, it is very useful to use not only Alb and CRP but also GPS.

Though Kim et al. reported that CRP-based prognostic scores (CRP, Glasgow Prognostic Score < GPS >, modified GPS (25) and Prognostic Index (26)) could not predict outcome in patients with UTUC in their cohort (27) and Ku et al. reported that albumin has superior prognostic value than other scores based on CRP (Glasgow prognostic score, modified Glasgow prognostic score, and prognostic index) (28), Inamoto et al. mentioned the impact of UTUC in the same way as us (29). The aim of this study was that preoperative GPS was significantly useful for prognosis evaluation in patients with UTUC.

The incidence of UTUC has increased during the past 20 years in the USA, and the prognosis for UTUC is generally poorer than that for bladder cancer (12). Prognostic predictors with a high specificity for UTUC are therefore needed to select the optimal treatment. In other words, it may be possible to consider adjuvant chemotherapy or other adjuvant optimal therapy with GPS as an indicator. That is what we are researching now. And at present, we consider the patient to be more carefully observed when considering GPS. However, clinically effective prognostic predictors have not yet been established as for other cancers, such as PSA for prostate cancer and AFP or hCG for testicular tumors. The reported prognostic predictors for UTUC include histopathological T stage, histopathological grade, tumor location, LNI, lymphovascular invasion (LVI) and surgical procedures. However, all of these are postoperative factors. Urinary levels of nuclear matrix protein 22 (NMP22), bladder tumor antigen (BTA) and urine CYFRA 21-1 have been investigated along with TPA, TPS and other substances (30), but the urinary levels are known to increase in the presence of urinary tract infection, making them unreliable as prognostic predictors. Thus, the only reliable preoperative prognostic predictor reported to date is serum CRP and serum CYFRA 21-1 (12). There were only 74 cases in total, and to the number of predictors put in the regression model because of the limitation, lymphovascular invasion, surgical procedures, T stage, grade, tumor location etc. were not included as predictors in this study.

In this study, ROC analysis, AUC and 95% CI were calculated, the cut-off value of CRP was 1.0mg/dL, and the cut-off value of CYFRA 21-1 was 6.0ng/mL.

Each of age (p=0.04), CRP (p=0.001), CYFRA 21-1 (p < 0.001) and GPS (p < 0.001) were statistically correlated with overall survival rates.

It is also attractive if GPS can also play the role of biomarker, which directly affects the disease state and volume of the tumor. In this study, overall survival is regarded as an endpoint, but we plan to examine the relationship with progression free survival.

In conclusion, our study demonstrated that GPS serves as an independent prognostic indicator for UTUC. This shows that treatment of UTUC may be able to select optimal treatment including kidney-sparing surgery. However, since there were only 4 cases in Group-3, there is a possibility that the analysis result may be influenced.

## STUDY LIMITATIONS

The present study is not without limitations. In this study, we analyzed all patients who were submitted to surgery in 26 years. Though total number of cases was 74, and 50% of the patients died, it should be analyzed with more increasing cases.

The authors have no conflicts of interest directly relevant to the content of this article.

## ABBREVIATIONS

CRP: C-REACTIVE PROTEIN
CYFRA 21-1: Cytokeratin-19
GPS: GLASGOW PROGNOSTIC SCORE
UTUC: UPPER URINARY TRACT UROTHELIAL CARCINOMA
UC: Urothelial carcinoma
PSA: prostate specific antigen
AFP: α-fetoprotein
hCG: human chorionic gonadotropin
RNU: nephroureterectomy
CSS: cancer-specific survival
NLR: neutrophil-to-lymphocyte ratio
SD: standard deviation
95% CI: 95% confidence intervals
ROC: Receiver operating characteristics
AUC: area under the curve
Alb: Albumin
IL: interleukin
LVI: lymphovascular invasion
NMP22: nuclear matrix protein 22
BTA: bladder tumor antigen
WBC: white blood cell
